# The Tension-Twist Coupling Mechanism in Flexible Composites: A Systematic Study Based on Tailored Laminate Structures Using a Novel Test Device

**DOI:** 10.3390/polym12122780

**Published:** 2020-11-24

**Authors:** Julia Beter, Bernd Schrittesser, Gerald Meier, Bernhard Lechner, Mohammad Mansouri, Peter Filipp Fuchs, Gerald Pinter

**Affiliations:** 1 Polymer Competence Center Leoben GmbH, Roseggerstrasse 12, 8700 Leoben, Austria; Bernd.Schrittesser@pccl.at (B.S.); Gerald.Meier@pccl.at (G.M.); Bernhard.Lechner@pccl.at (B.L.); Mohammad.Mansouri@pccl.at (M.M.); PeterFilipp.Fuchs@pccl.at (P.F.F.); 2 Department of Polymer Engineering and Science, Montanuniversitaet Leoben, Otto Gloeckelstrasse 2, 8700 Leoben, Austria; Gerald.Pinter@unileoben.ac.at

**Keywords:** flexible composite, fiber-reinforced elastomer, load-coupling mechanism, bending-extension coupled structures, extension-shear coupling effect

## Abstract

The focus of this research is to quantify the effect of load-coupling mechanisms in anisotropic composites with distinct flexibility. In this context, the study aims to realize a novel testing device to investigate tension-twist coupling effects. This test setup includes a modified gripping system to handle composites with stiff fibers but hyperelastic elastomeric matrices. The verification was done with a special test plan considering a glass textile as reinforcing with different lay-ups to analyze the number of layers and the influence of various fiber orientations onto the load-coupled properties. The results demonstrated that the tension-twist coupling effect strongly depends on both the fiber orientation and the considered reinforcing structure. This enables twisting angles up to 25° with corresponding torque of about 82.3 Nmm, which is even achievable for small lay-ups with 30°/60° oriented composites with distinct asymmetric deformation. For lay-ups with ±45° oriented composites revealing a symmetric deformation lead, as expected, no tension-twist coupling effect was seen. Overall, these findings reveal that the described novel test device provides the basis for an adequate and reliable determination of the load-coupled material properties between stiff fibers and hyperelastic matrices.

## 1. Introduction

The demand for customized products that are tailored to meet specific requirements is continually growing. Due to the increase in efficiency, weight reduction and performance, lightweight designs are receiving increased interest in numerous applications. Hence, conventional materials are reaching their application limits, which creates the need to focus on multi-material solutions [1,2]. Especially for elastomers, the additional integration of reinforcing structures has already led to promising concepts enabling higher bearable loads while good flexibility, damping and absorption performance are still retained [3]. This approach has been successfully applied in the industry such as automotive tires [4], conveyor belts [5] or fiber-reinforced elastomeric seismic isolators [6,7]. Advancing from traditional composite, the basic idea of “learning from nature”, e.g., nacre mimetic nanostructures [8] or staggered model [9], is also pursued in the design of new composite structures. The implementation of methods, designs, and processes from nature with suitable transfer criteria into various fields of engineering is described as biomimetics [10]. Recent scientific approaches have demonstrated interesting concepts by implementing fiber-reinforced elastomers with distinct hyper-elasticity as so-called smart materials. The concept of these soft matter applications can be found in the field of aerospace or automotive industries as aeroelastic wings [11,12] with the ability to serve multiple functions for optimized aerodynamic performances. Another aspect for the usage of morphing structures are in soft robotic applications such as exoskeletons [13] or artificial fingers [14], where sufficient strength combined with significant large deformations have to be ensured. The crucial challenge is to apply the right material, fiber-matrix material combination and resulting functionality in order to find the best solution. Especially for soft matter applications, the combination of elastomers with controlled oriented reinforcement can generate advanced composites with distinct direction-dependent properties. Whilst the acting energy can be merged or the resulting force redirected, no damage is initiated and the energy might even be used favorably [14,15,16]. The state of the art regarding current developments for stimuli-responsive materials with load-coupling effects enhanced by external triggers can be structured into four classes relying on: (i) pressure (pneumatically or hydraulic) [17,18], (ii) electrically [19,20], (iii) temperature [16,21] and (iv) mechanically [22,23] initiated deformation. Most of the work has been done on pressure or temperature triggered load-coupled effects, which are often hybridized with an electrical trigger [24,25,26], except if the electricity is not exclusively implemented as e.g., piezoelectric generated effect to enable shape-memory effects [27,28]. However, the focus is mainly put on the demonstration and feasibility of demonstrators [29,30], whilst the mechanical properties related to structure-property interactions, especially for microstructure analysis, are considered in a limited way [31,32]. Therefore, profound knowledge and an adequate quantitative investigation regarding the performance and mechanical behavior of fiber reinforced elastomers combined with an external trigger is necessary. Since the ability of load transfer between the fibers and the matrix is crucial to analyze load-coupling mechanisms, a tailored fiber orientation as well as an optimized interfacial fiber-matrix bonding is indispensable to ensure an adequate adhesion with an intended load-coupling [23,33]. 

The classical laminate theory (CLT) as a material law for the prediction of stiffness and stress in multilayer composites offers a well-established method to quantify stress-deformation couplings of composite materials numerically [34,35]. Since this material law is based on Kirchhoff’s plate theory [35], simplifications and boundary conditions, such as linear elasticity and ideal composite conditions, are unavoidable [23,36]. Extensive studies including the CLT for thermoset-based composites have already been carried out. However, this material law cannot be transferred directly into flexible composite materials, which possess a significant textile-like behavior [22,37]. Compared to stiff matrices, fiber reinforced elastomers show further beneficial aspects regarding the damage performance by showing a significant broader motion range, especially when it comes to bending or twisting coupled behavior [34]. Due to the hyperelastic matrix, the distinct greater mobility of the embedded fibers can induce local stress concentrations by out-of-plane wrinkling, which results in folding or buckling. This behavior may lead to a local fiber-matrix debonding at micro scale without resulting into a complete failure or premature composite breakage induced by delamination like for thermoset-based composites [38,39,40]. Recent studies on soft morphing structures with anisotropic properties using bend-twist or bend-extension coupling described the need of a modified formulation of the CLT, as stiffness and strength of fibers and elastomers differ significantly [34,37,41]. Subsequently, the right choice of the test device with a corresponding setup is crucial to determine exact material properties of highly flexible composites. Most of the existing measurement devices for fiber reinforced composites are limited to thermoset-based matrices, which cannot fulfill the required test conductions due to local stress concentrations, slippage or pre-damages by inappropriate clamps [38,42]. These test methods related to thermoset-based composites and their load-coupled properties assume that clamping induced compressive stresses can be neglected [41,43]. In this context, additional tabs with a certain tab taper angle are typically considered for a better load transfer and uniform deformation distribution during the test. However, those are not applicable for fiber reinforced elastomers due to the high necking, which leads to an interface release between sample and the tab [44,45]. As the stiffness and strength of fibers and the elastomer matrix differ significantly, the mechanical properties are much more complex to determine and crucial for evaluating the fiber-matrix adhesion [1,46]. Moreover, to trigger load-coupling mechanisms in flexible composites effectively, an in-depth knowledge of the fiber-matrix bonding and mechanical performance of single-fiber, fiber-bundle and simple composite structures within the principle of a test chain constituting the aspects from model- and component level are required [47]. Recent studies on fiber reinforced silicones were carried out focusing on the properties of the fiber-matrix interface and reported on the challenge of dealing with hyperelastic elastomers [48].

The aim of this research is to investigate the mechanical properties of tailored fiber-reinforced elastomers triggered by tension-twist coupling effects. A newly developed test setup is designed to avoid negative clamping or other influences e.g., local stress concentrations, slippage or premature failure, whilst providing a convenient, fast and reliable method. Since endless fibers encapsulated in elastomer increase the complexity for the material characterization, the main challenge is to overcome the hyperelasticity, which implies a limited inherent stiffness. One focus was on the verification of the new load-coupling test device to study the distortion stresses and twisting induced by an external force. Thus, several parameters and their influence on the load-coupling were analyzed by a test plan including fiber orientation, lay-up and stacking sequence. Based on this, the possibility to obtain the indicating parameters of maximum tension force and torque including the associated twisting was proven. The generated customized material parameters provide the basis for further numerical elastic body simulations based on well-established composite material laws, e.g., CLT, and to emphasize tailored performance predictions. 

### Theoretical Background for Load Coupled Structures and Design Principles

In general, flexible composites or so-called smart materials, are designed by combining the characteristics of anisotropic materials with soft morphing structures [37,41]. By exploiting these structure-properties, the interaction of different components is indispensable considering the rigid fibers as anisotropic reinforcement, the soft matrix and the mandatory trigger e.g., mechanical or pneumatical initiated as an external stimulus. The interaction of all individual elements in total generates and quantifies the intended load-coupling mechanism [34]. The stiffness towards twist as well as the in-plane shear strength of the flexible composite related to different directions is controlled by the force transmission of the anisotropic oriented fibers versus the viscoelastic behavior of the matrix [33,49]. Hence, the CLT represents a computational tool to describe overall deformations. These findings on load-coupled effects can be integrated featuring any combination of in-plane deformation, out-of-plane deformation, and twisting in the flexible composites. Following the purely formal derivation of the CLT, this can be written according to Equation (1), where the layer structure is basically described by the material law of the single layer. Thus, a correlation is established between the internal forces **n** and moments **m** of the laminated plies, the elasticity parameters constituting the layer built-up, and distortions **ε** as well as curvatures **κ** of the intermediate surfaces inside the composite [34,35].

(1) $$\left[\;\begin{array}{c} n\\ m \end{array}\;\right]=\left[\;\begin{array}{cc} A & B\\ B & D \end{array}\;\right]\left[\;\begin{array}{c} \varepsilon \\ \kappa \end{array}\;\right]$$

**Figure 1:**
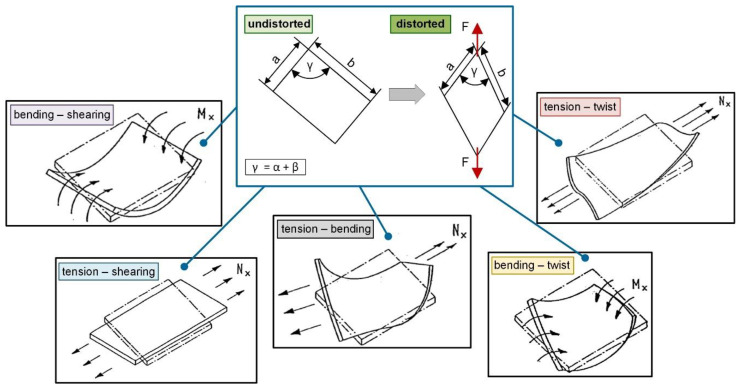
Schematic illustration of distortion processes (undistorted versus distorted) and corresponding deformation possibilities of bending, shearing, twist or tension depending on the load-coupling related to the material’s law for fiber reinforced composites [35].

The combination of matrices A, B and D is also known as the stiffness matrix K. For different occurring extents of symmetry of material properties depending on the stress-strain relationship and the corresponding anisotropic composite structure, the subsequent reduction in the number of elastic constants in the stiffness matrix needs to be considered [35]. In this context, the determination of the material data parameters with a new test device have to fulfill these requirements. Matrix A equals the strain stiffness (in-plane moduli) connecting the load transfer with the distortion of the intermediate surfaces and thus, contains the elasticity law that connects in-plane loads to in-plane strains. Furthermore, matrix D represents the bending stiffness matrix, which links the moments of elongation with the curvatures of the intermediate surfaces. Matrix B is consequentially combining the curvatures of the intermediate surfaces with the normal and shear force transition, whilst the distortions of the mid-surfaces is linked to the intersection moments and is referred as the matrix of coupling stiffness [34,35]. Due to this, the load transmission leads to specific distortion of the intermediate surfaces as well as to associated curvatures (also in reverse conclusion). A graphic illustration of the deformation concept versus subsequent distortion possibilities is schematically shown in Figure 1. For composite materials with a pronounced flexibility, further challenges occur, which are unavoidable and inherent to the material characteristics and can lead to considerable influences, such as trellis effects and maximum locking angle induced wrinkling. These phenomena have been investigated in detail in previous studies based on quasi-static tensile tests [45,47]. The results reveal a correlation between the maximum bearable in-plane shearing until out-of-plane shearing (wrinkling or the trellis effect) occurs. The main reason for this is the deformation obstruction due to the conventional rigid clamping system of the used test setup, which suppresses a twist and inevitably leads to the trellis effect [50]. Using a movable or semi-movable test device, these generated deformation-induced stresses will be relieved within a twist or bending motion, which results in a load-coupling mechanism [35]. 

## 2. Materials and Methods 

### 2.1. Reinforcement 

Commercial E-type glass fibers used in this study were provided from CS Interglas AG (Erbach, Germany) with a twine thickness in warp and weft-direction of about 68 tex. A textile from a single batch and with a plain weave exhibits an area bundle distribution of 50/50 in the 0°/90° direction yielding an area weight of 220 g/m^2^ ± 5%. The filaments comprised a mean diameter of 10 µm and were modified with a standard industrial silane-based surface sizing (FK144). Moreover, the mechanical properties of glass fibers were investigated by standardized tensile tests according to ASTM D2256 [51] using a universal testing machine (Series 5500, Instron GmbH, Darmstadt, Germany). The tests were performed with the settings of a 1 kN load cell, a free gauge length of 250 mm and a crosshead speed of 300 mm/min including a pneumatically controlled mandrel type clamping system for fixing the fibers. Additional rubber pads were required to protect the fibers from any pre-damage in the clamping area as well as a preload of 1 N to ensure identical initial test conditions. 

### 2.2. Matrix Material

Elastosil RT601 A/B was used as matrix material and was obtained from Wacker Chemie AG (Munich, Germany). Elastosil RT601 A/B is a hyperelastic two-component polydimethylsiloxane (PDMS), (the prepolymer, part A and crosslinking system, part B comprising a platinum catalyst). Based on the characteristic inorganic structure and organic groups with siloxane units, PDMS represents a good intermediate position between inorganic and organic compounds. Due to the exploitation of these structural properties, the stronger bonding energy of PDMS in combination with glass fibers (GF) results in an enhanced interfacial adhesion, which consequently influences the flexible composite properties, particularly for the investigation of load-coupling effects. Thus, further effects caused by fillers or material morphology can be reduced. For the formation of the PDMS network, a mixing ration of 9:1 (part A: part B) was applied. According to the manufacturer’s recommendations, the uncured elastomer formulation degassed under vacuum to avoid any air bubbles or inclusions. The curing and polymerization were carried out at 70 °C for 60 min in an air circulating drying oven. Furthermore, the mechanical properties of PDMS were evaluated in standardized uniaxial tensile tests according to ISO 37 [52] with the corresponding specimen geometry of type 2 by utilizing a universal testing machine (Z010, Zwick Roell GmbH & Co. KG, Ulm, Germany) equipped with a 500 N load cell. The gauge length was set to 50 mm with a measuring length of 20 mm and a test speed of 10 mm/min including a pneumatically controlled clamping unit. 

### 2.3. Shear Stresses Coupled by Fiber Orientation

**Figure 2:**
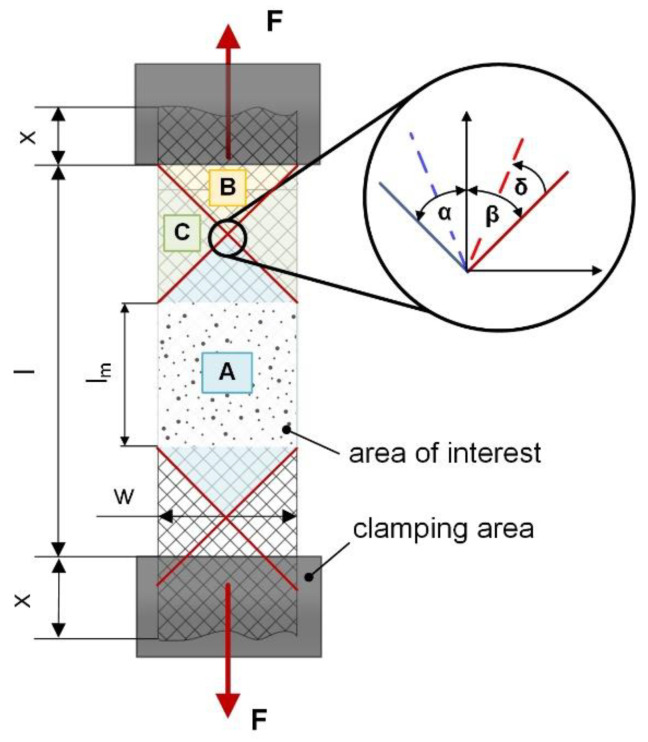
The three main shear zones (A, B and C) corresponding to the exemplary ±45° fiber orientation in a composite tensile test configuration [44] and the explanation of the in-plane shearing effect with the shear angel δ on undistorted (blue and red line) and distorted (dashed blue and red line) fibers [47].

In particular, during the deformation process of fabrics, shearing is an important influencing factor, which is strongly affected by the fiber orientation and thus, emerges whenever the orientation differs from the loading direction of the external acting force. In this context, three main zones (A, B and C) occur in the textile through an applied load, which obtain significant different deformation modes and are schematically illustrated in Figure 2 [44,53].

The area of zone A comprises no impaired fibers, since all fibers are unaffected by grips and only connected via weave points (between warp- and weft yarns) by the surrounding elastomeric matrix whilst the necessary adhesion is achieved by the matrix and supported through e.g., friction or ondulation effects. Thus, this region is defined as clamping stress free and deformation is only constituted by the present shearing [53]. In contrast to this, zone B is not deformed due to the clamping, where fixed fibers are hindered in shearing. However, zone C contains partially constrained and unfixed fibers, which represents a sort of mixed type of shear and elongation [44]. Based on this, composites with strong textile behavior lead to pronounced out-of-plane deformation after exceeding a certain threshold value (locking angle) [44,54]. This causes wrinkling especially in rigid test, since bending or twisting motion is inhibited during progressive deformation. This effect is termed as the trellis effect which is related to the fiber orientation and the composite lay-up [54]. The in-plane and out-of-plane shearing versus the trellis effect in fiber reinforced elastomers was investigated in detail in previous studies [47].

### 2.4. Specimen Preparation and Test Method

For better comparability and to minimize any negative influences concerning the manufacturing of the composite test specimens, a vacuum resin infusion (VARI) process was chosen [33,47]. The maximum feasible pressure during the impregnation and consolidation phase was set with about 0.1 MPa (atmosphere pressure) [2,55]. The used mold release agent (Mono-Coat 1625W) was provided by Chem-Trend GmbH (Maisach, Germany). The cutting step of the reinforcement layers was carried out with a professional cutter (G3 M-1600, Zünd Systemtechnik AG, Altstaetten, Switzerland), which is additionally equipped with vacuum table to minimize fiber undulations or drape defects. Due to the high viscosity of the uncured PDMS besides the presence of vacuum, permeable lines, flow help and a perforated release film are mandatory to achieve a good laminate quality. After the curing step, the GF-PDMS composite plates were demolded and rectangular specimens were prepared with the cutter. All samples consist two layers of reinforcement regardless the implemented fiber orientation in the subsequent tests. 

Based on previous studies [47] focusing on tailored fiber-reinforced elastomers with different fiber orientations and their influence on structure-property interactions and adhesion properties, composite tension tests with a specific width to length ratio were used to analyze the in-plane shearing. The results showed a significant effect caused by different fiber orientations and obtained that an out of plane deformation starts to occur sooner if the textile is unbalanced related to stresses. This leads to distortion inhibitions and further to load-coupling effects. In order to establish a definite comparability with the conducted tensile tests, the width to length ratio of 1:3 is implemented for these tests [47]. In this context, the gauge length is defined as the distance between the clamps in the testing machine and set to 45 and 90 mm. The measurement length corresponds to 20 mm and was recorded optically due to the textile-like behavior. 

**Figure 3:**
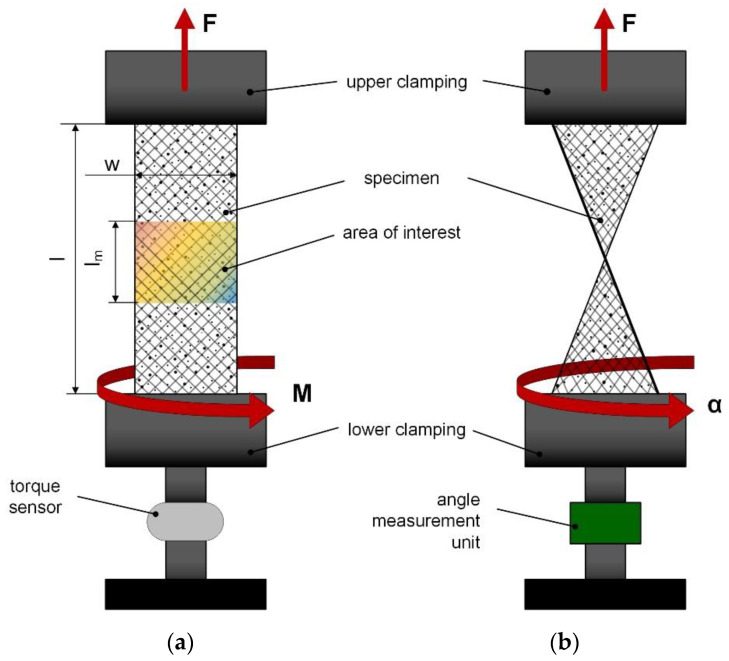
Schematic measurement procedure of tension-twist load coupling test principle on glass fiber-polydimethylsiloxane (GF-PDMS) composite specimens in fixed mode (**a**) for measuring the torque M and in movable mode (**b**) for measuring the twist angle α triggered by external tension force.

To determine load-coupling mechanisms in flexible composites, a new test setup was developed to accommodate the high flexibility as well as to be able to implement the test device in conventional testing machines (see Figure 3). Furthermore, this device has to prevent slippage or clamp-induced damage during the tests to avoid further stresses which leads to misleading results and premature material failure. Conventional pneumatic grips with one side closing function (Zwick Roell GmbH and Co. KG, Ulm, Germany) are not suitable and a modified clamping system had to be considered. Thus, another challenge is the accurate specimen position to avoid any negative effects related to tilting or asymmetrical stress distribution. Therefore, the modified clamping system is able to prevent this mechanism and ensures a loading situation self-aligned along the machine axis. The developed test setup is designed in order to measure a torsional moment in fixed mode and a twist angle in rotating mode. In this context, sensors are an essential tool for data recording to adequately describe these two states. Subsequently, corresponding measuring units are implemented using a torque sensor 9339A provided by Kistler Group (Winterthur, Switzerland) with a designated measuring range between −10 Nm and 10 Nm and an angle sensor AEDB-9340 series from Avago Technologies (Broadcom Inc, San Jose, CA, USA). This novel test setup is replacing the lower clamping unit, whilst the upper clamping comprises the standard pneumatically driven grips from Zwick enhanced additionally with a special fixing unit [42] especially for fiber reinforced elastomers. Due legal reasons, any further information or visual illustration of the test setup cannot be provided regarding the current patenting process, since this presented assembly is a special concept that is designed to switch between fixed and rotating mode enabling measurements of torque and twist including a modified clamping system, which ensures sufficient clamping especially for such flexible composites. 

All tests were performed at standard atmosphere conditions according to ISO 291 (20 °C, 50% r.h.) [56] with a constant displacement rate of 10 mm/min. The tests were conducted with a universal testing machine (Zwick Roell GmbH and Co. KG, Ulm, Germany) equipped with a 10 kN load cell. The composite specimens were deformed to a maximum elongation of 20% in order to ensure an almost unaffected fiber-matrix bonding, since in zone A the load is transferred only by shearing [57] in the weave points of the textile and Elastosil RT601 A/B shows linear elasticity until approximately 40% deformation [55] and thus a reliable load-coupling is provided. For the subsequent data interpretation, the average value of three specimens for each setting was calculated. To ensure equal test and the same initial starting conditions during the experiments, a preload of 1 N was considered.

For the evaluation of the load-coupling, further effects of viscoelasticity in flexible composites [33,49] are reduced by the constant set test speed. Due to that, a methodically validated test plan was developed, where the fiber orientation was set depending on the considered reinforcing structure. Therefore, the orientations ±45°, 30°/60° and a combination of both 30°/60° and ±45° (henceforth written as 30°/60°//±45°) related to previous findings on numerical simulation models [58] were chosen for the composite lay-up with textile. In order to determine the maximum bearable twist angle and corresponding torque, both gauge lengths were considered in addition due to the high flexible behavior of these composites, since the form stability and the distinct textile-like character can be influenced significantly.

### 2.5. Optical Analysis

Supplementary optical analysis including light microscopy of the composite samples to support the comparability and interpretation of the performed tests was performed prior to (as reference purpose) and directly after the load-coupling test to prove the unaffected fiber-matrix interface and to avoid further environmental influences. Regarding the pronounced elasticity of the flexible composite as well as to record deformation behavior in three dimensions, the strain ratio and twist was measured together with a digital image correlation system (Prosilica GT 6600, Allied Vision Technologies GmbH, Stadtroda, Germany) along with the implemented sensors. Moreover, a sprayed pattern was used on the sample surface to achieve a higher accuracy in data. 

## 3. Results and Discussion

In previous work, promising and reliable results were achieved using a modified test setup for tension tests on flexible composites to investigate the feasibility as well as the effect of different fiber orientations on wrinkling caused by trellis effects [47]. Based on these findings, the following study focuses on the realization and verification of a new test setup to determine tailored tension-twist coupling mechanisms in flexible composites. Therefore, a suitable step-by-step transferability based on the test chain concept and its transfer criteria (from model to component-like level) was developed whilst enabling a quantitative understanding and clear validation. With this approach, the influence of specific stiff reinforcing fibers on hyperelastic elastomers and their load-coupled behavior could be studied in detail, within the findings from the single component materials, the fiber-matrix material combination and shearing behavior due to analyzed tension tests already considering different reinforcing orientations. The applicability of the presented load-coupling test setup was assessed to determine the twist angle as well as torque and to get a deeper insight into the mechanical properties of flexile composites.

### 3.1. Reinforcement, Matrix and Composite

The mechanical properties of the constituents (fibers and matrix) are compared in Table 1. The results for PDMS revealed an elongation at break of about 108.6% with a determined stress at break of about 4.5 MPa. Thus, an elongation at break of approximately 1.4% with a corresponding force at break of about 119.8 N is given for the glass fibers of the textile. 

**Figure 4:**
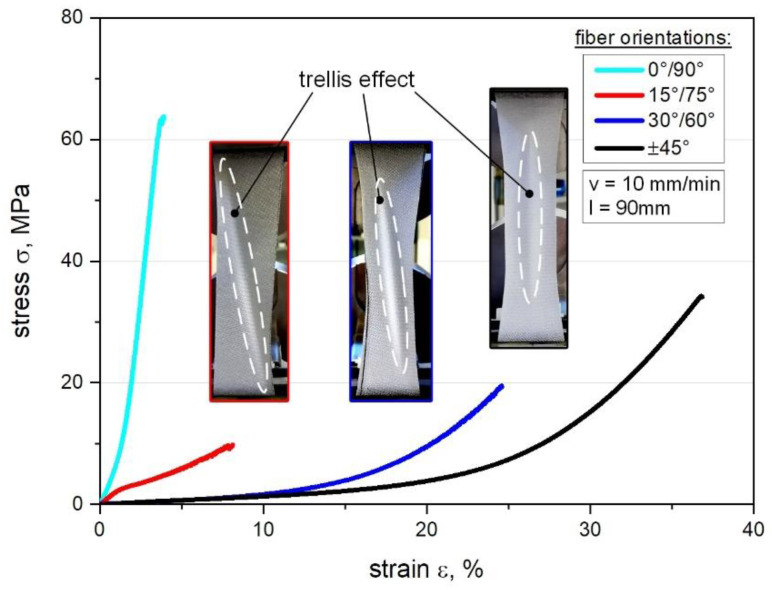
Comparison of stress-strain curves of the different fiber orientations (±45°, 30°/60°, 15°/75° and 0°/90°) for the GF-PDMS composite with textile reinforcement as obtained from composite tensile tests with the accompanying trellis effect related to the wrinkling and out-of plane shearing.

Former investigations based on quasi-static tensile tests were conducted to investigate the mechanical properties of tailored fiber-reinforced elastomers, which are added as supplemental information to complement the results (see Figure 4). In this context, the influence of different fiber orientations (±45°, 30°/60° and 0°/90°) versus the deformation behavior, the shear-induced in-plane distortion and maximum possible locking angle (resulting in the trellis effect) were analyzed [59]. The results reveal that shearing becomes increasingly dominant depending on how significantly the fiber orientation deviates from the loading direction. Therefore, the ±45° fiber oriented composite material has a higher flexibility (matrix dominated performance) with an elongation at break of about 38.2% ± 0.5 with a stress at break of about 34.3 N/mm^2^ ± 2.9 compared to the configuration of e.g., a composite configuration with 0°/90° fiber orientation (fiber dominated). As expected, the stiffness can be enhanced particularly at small strain rates between 0% to 10% but also with a clear reduction in flexibility, which is directly correlating with the maximum locking angle. Since the use of elastomers such as PDMS as alternative matrix for composites, the trellis effect is amplified compared to typical thermoset-based composites [44,57] due to the textile-like behavior [33]. 

### 3.2. Tension-Twist Coupled Effect

**Figure 5:**
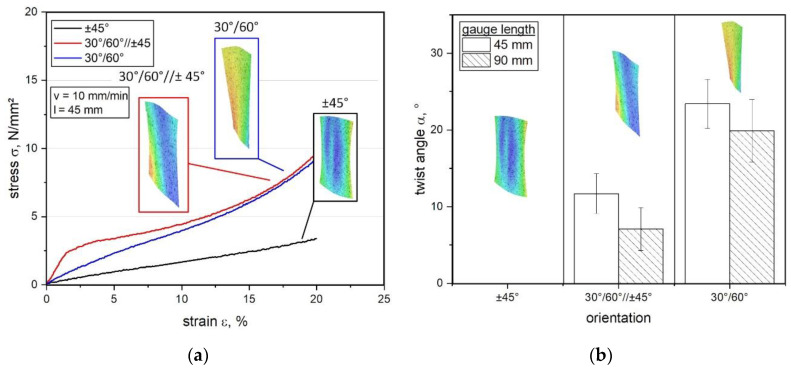
Stress-strain curves obtained from tension-twist load-coupling tests for GF-PDMS composites with textile reinforcement (**a**) and comparison of different gauge lengths versus inherent stiffness on the twist angle α performed with different fiber orientations (**b**) including digital image correlation in false color display.

Regarding the load-coupling test device, the stress-strain curves for tension-twist coupling measurements with 45 mm gauge length are illustrated in Figure 5a representing all three different fiber orientations. Due to the expected behavior, the results of the ±45° oriented composites reveal a significant matrix dominated performance, which can be compared with the tension tests given in Figure 4. This can be explained by the behavior of the warp and weft yarns, which starts to shift during deformation and the yarns are able to undergo larger displacements until the fibers converge with the loading direction [53]. Related to this, the 30°/60° composite shows a steeper slope, which can be attributed to the increased fiber dominated properties leading to a higher stress of about 10 N/mm^2^ (approximately 40%) than for ±45° composites at the same strain level of 20%.

In contrast to that, the special configuration of 30°/60°//±45° composite as mixed type indicates a significant higher stress-strain slope at the beginning compared to the other settings. However, this trend tends to converge with increasing deformation, so that the material behavior approximates more closely to the 30°/60° composite, which is visible in Figure 5a. In this context and contributed to the conducted pre-simulations this mixed configuration has an intermediate position regarding the predicted twist versus torque load-coupling behavior [23,41]. Moreover, the results show that the stiffness differs, especially at small strain ratios from 0% up to about 10% deformation. In particular the highest stiffness is observed for the mixed configuration, which can be explained by the interaction of the two differently oriented layers creating further locking effects in terms of several effect of shearing during the deformation [44,47].

Thus, the results for twisting and corresponding torque versus different fiber orientations are reported in Table 2, investigating an increase of the twist angle as well as torque from ±45° composites up to 30°/60° composites, which shows the highest maximum twist angle of about 24.4° and torque of about 82.3 Nmm. As expected, the ±45° composite reveals no twist or torque, which can be explained by the symmetric lay-up whilst the wrinkling is occurring parallel to the loading direction (see Figure 5a) [60]. Generally, this behavior proves that shearing becomes increasingly dominant according to the degree of deviation between the loading direction and fiber orientation [44,61]. Therefore, the effect of in-plane shearing during deformation is mainly influenced by the fiber orientation, since the displacement of fibers relative to each other reaches a maximum deformation angle (in-plane) at some point and the flexible composite starts to wrinkle [47,59]. This locking angle appears sooner in terms of asymmetric composite lay-ups causing an unbalanced stress state so that out-of plane deformation leads to a twist based on load-coupling [45,54]. However, if this decisive rotating mechanism is inhibited, torque is inevitably generated. Besides, all reported measurements for torque and twist angle show generally higher standard deviations, which are currently under investigation and compared with accompanying simulations. In this context, those preliminary analysis cannot be attributed unambiguously to the test setup, since in accordance to the recent findings several different influences are included such as specimen production influences, specimen handling or inherent rigidity of the test setup. Due to that, the results of this study represent initial measurements within the focus on the principal realization and feasibility of a potential test device, so that those effects are investigated in detail in ongoing studies.

For determining the load-coupling behavior due to the influence of different parameters of gauge length and fiber orientation versus the maximum reachable twist angle α, the results of GF-PDMS composites are displayed in Figure 5b. To provide a better overview, the influence of various clamping lengths versus maximum twist angle α (descripted in Figure 3) is considered in a quantitative manner. As expected, the results demonstrate a significant increase of α when reducing the gauge length from 90 mm to 45 mm. A reason for this is the fact, that the inherent stiffness of such highly flexible composites can be clearly increased due to this adjustment, especially in combination with PDMS matrix [62,63]. Regarding the mixed configuration of 30°/60°//±45° composite, the results indicate no clear effect in the twist angle although a significant increase in stiffness can be observed in the stress strain curve especially at the beginning (see Figure 5a). This could be due to the fact, that the already achieved deformation was too small to reach a sufficient influence on twisting, which is probably also supported by the hyperelasticity of the matrix. However, the standard deviation tends to increase with higher gauge length, which could be due to the fact of the hyperelastic matrix as well as for the higher bearable elasticity by the fibers. Moreover, the quantitative comparison of the maximum twist angle versus the fiber orientation shows a good agreement with the stress-strain curve obtained from measurements with the presented load-coupling test device. Generally, it is evident, that a reduced fiber angle from ±45° to 30°/60° results to an enhanced stiffness and thereby to higher tension stresses at equal strain level e.g., at a strain of 20%. However, these findings demonstrate that despite an enhanced stiffness, the flexibility (imparted by the PDMS matrix) is still retained even with a more fiber dominated 30°/60° composite. This has a positive influence on the fiber-matrix interface and thus, for the load-coupling mechanism [33]. Based on this, further experiments need to be carried out to investigate and to verify the measurement sensitivity varying the fiber-matrix adhesion and thus, the load transfer by tailored fiber surface treatments which influences the interface and load-coupling effect. Furthermore, the influence of different reinforcing types is currently under investigation, how fiber-fiber friction, reorientation of fibers by extension towards fiber angle changes or undulation affects the load transfer in textiles compared to mats, clutches and prepregs. Subsequently, further experimental investigations are analyzed in ongoing studies and compared with simulations related to load-coupling predictions with the CLT.

### 3.3. Optical Damage Analysis

**Figure 6:**
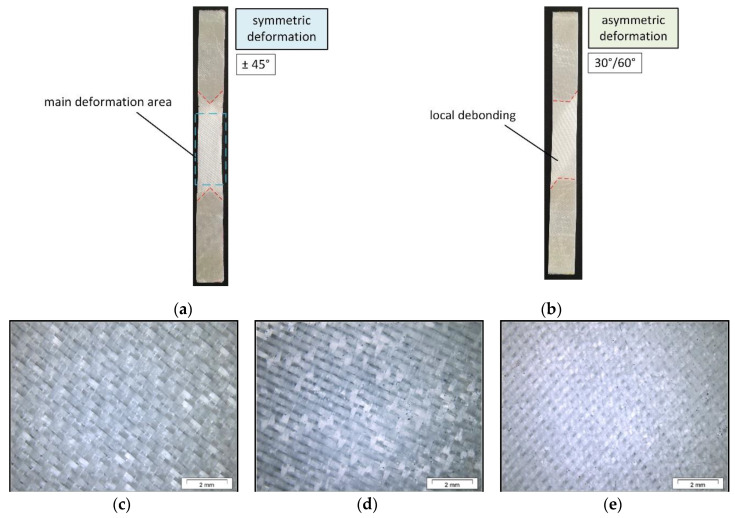
Optical damage analysis of GF-PDMS composites from tension tests with ±45° fiber orientation (**a**) and 30°/60° (**b**) oriented compared tension-twist load-coupling tests with ±45° composite: in initial state (**c**), after load-coupling test (**d**) and after tension tests (**e**).

To ensure an unaffected fiber-matrix adhesion with no visible local debonding and thus an adequate load transfer, light microscopy images were taken from the GF-PDMS composite samples. Besides the results obtained from the tension-twist coupling and simple tension tests, the accompanying optical damage analysis show further information about the test performance providing a good comparability with the mechanical measurements [64]. As illustrated in Figure 6a,b, the results on pure tension tests according to the ISO 527-4 [65] reveal that a symmetric deformation is occurring for ±45° composite following the affiliated wrinkling shape during deformation [59,66].

Compared to this, the 30°/60° fiber-oriented composite clearly indicates an asymmetric deformation accompanying with the wrinkling performance. Both configurations display local debonding in the interface at the main deformation area, which can be recognized by the shift of the light refraction due to the separation process [64]. This finding can be further confirmed in more detail with a corresponding light microscopy image (see Figure 6e). Further, despite the debonding, a complete failure of the entire sample is not generated. In contrast to that, the optical damage analysis of the load-coupling tests (see Figure 6d) shows an undamaged and still good fiber-matrix adhesion compared to the original initial state (see Figure 6c), respectively. Therefore, it can be assumed that the adhesion is unchanged, which verifies a successful load transfer via the presented load-coupling test setup.

## 4. Conclusions

Since research interest on so-called smart materials is continuously growing, this work mainly focuses on the demonstration and feasibility of a new test device intended to provide the basis for an adequate analysis of tension-twist mechanisms triggered by an external mechanical force in specific flexible composite with tailored fiber orientation. The aim of this research is to investigate structure-property interaction towards load-coupling effects to obtain material data including customized twist and torque properties especially for morphology analysis as well as to implement these findings in constitutive numerical approaches, in simulation models including well-established composite material laws. Due to the high flexibility, silicone in combination with glass fibers as the considered single components were chosen to demonstrate a selected fiber-matrix combination as well as the fiber orientation to achieve a reliable direction-dependent characterization accompanying anisotropic composite performance. Within the focus, a quantitative study including a methodical test plan was elaborated to study the impact of various influencing parameters and to assess their effects on the twist and torque.

The results of the tension-twist coupled tests reveal that the mechanical properties related to the structure-properties and thus the load-coupling effect can be optimized in a specific manner depending on the fiber orientation and the composite lay-up. Furthermore, the stiffness can be controlled and improved without impairing the flexible properties of the silicone significantly, whilst the fibers are reinforcing the composite. Additional studies are currently under investigation focusing on the impact and effect of different reinforcing types on the load-coupling properties. Moreover, further work on various treated fiber surfaces to influence the fiber-matrix adhesion and therefore the load transfer with tailored chemical surface modifications as well as different fiber-matrix material combination is already in progress. Related to this, more studies on the physical phenomena behind the developed load-coupling test device needs to be carried out. Overall, the results demonstrate that the presented test method improved by specific sensor systems shows a good agreement in combination with the optical damage analysis. The results of the load coupling tests reveal that even for small lay-ups the 30°/60° oriented composite with an asymmetric deformation triggers the highest torque of about 82.3 Nmm with a twisting angle of almost 25°. The mixed configuration of 30°/60°//±45° composite has an intermediate performance with a maximum achievable twisting angle of about 12°, since the ±45° composite with the highest deviation between fiber orientation and loading direction, as expected, undergoes no tension-twist coupling related to the symmetric deformation. Knowing this, the verification of the test device is validated, which allows sufficient accuracy of the material pre-analysis on tension-twist coupling mechanisms in a fast and easy way, whilst also contributing to a better understanding of the performance of fiber reinforced elastomers. Hence, this study evaluates the structure-property interaction of smart materials and highlights the essential contribution on the composite properties due to tailored load-coupling mechanisms. Moreover, further research should be carried out in terms of the comparability to macro scale performance between microstructure and application-like performances. This test method was developed to provide a base model to study load-coupling mechanisms adequately as well as a suitable link in a possible test chain between laboratory and industry applications.

## Figures and Tables

**Table 1:** Results for single component tests on GF and PDMS.

	GF	PDMS
max. force *F*_max_, N	119.8 ± 2.3	36.1 ± 1.1
max. strain *ε*_max_, %	1.4 ± 0.1	108.6 ± 8.6

**Table 2:** Results for GF-PDMS composite with glass fiber textile and 45 mm gauge length for different fiber orientations.

	Max. Twist Angle α, °	Max. Torque T, Nmm
±45°	0.0	0.0
30°/60°//±45°	11.7 ± 2.6	56.3 ± 9.6
30°/60°	24.4 ± 3.2	82.3 ± 11.1
